# A Smart Visual Sensing Concept Involving Deep Learning for a Robust Optical Character Recognition under Hard Real-World Conditions

**DOI:** 10.3390/s22166025

**Published:** 2022-08-12

**Authors:** Kabeh Mohsenzadegan, Vahid Tavakkoli, Kyandoghere Kyamakya

**Affiliations:** Institute for Smart Systems Technologies, University Klagenfurt, 9020 Klagenfurt, Austria

**Keywords:** optical character recognition, harsh real-world conditions, document analysis, deep neural network, robust OCR

## Abstract

In this study, we propose a new model for optical character recognition (OCR) based on both CNNs (convolutional neural networks) and RNNs (recurrent neural networks). The distortions affecting the document image can take different forms, such as blur (focus blur, motion blur, etc.), shadow, bad contrast, etc. Document-image distortions significantly decrease the performance of OCR systems, to the extent that they reach a performance close to zero. Therefore, a robust OCR model that performs robustly even under hard (distortion) conditions is still sorely needed. However, our comprehensive study in this paper shows that various related works can somewhat improve their respective OCR recognition performance of degraded document images (e.g., captured by smartphone cameras under different conditions and, thus, distorted by shadows, contrast, blur, etc.), but it is worth underscoring, that improved recognition is neither sufficient nor always satisfactory—especially in very harsh conditions. Therefore, in this paper, we suggest and develop a much better and fully different approach and model architecture, which significantly outperforms the aforementioned previous related works. Furthermore, a new dataset was gathered to show a series of different and well-representative real-world scenarios of hard distortion conditions. The new OCR model suggested performs in such a way that even document images (even from the hardest conditions) that were previously not recognizable by other OCR systems can be fully recognized with up to 97.5% accuracy/precision by our new deep-learning-based OCR model.

## 1. Introduction

Optical character recognition (OCR) systems are software systems that can convert document images into machine-readable text documents. Thus, hard-copy documents that previously needed to be manually entered or retyped by hand can now be automatically converted using digital OCR technology after they have been digitized by either scanners or cameras. OCR systems are widely used in various contexts, such as automatically archiving information [1], document authentication [2], handwriting recognition, symbol recognition [3], etc.

Today, digital cameras are widely utilized. However, when compared to traditional scanners, captured images from digital cameras (including those in smartphones) are not always particularly good when collecting document images with the required quality in terms of optimal performance of either OCR systems [4,5,6,7] or document-image-based document classification systems [8,9]. Document images captured using digital cameras (eventually embedded into smartphones) are typically degraded by various distortions, such as noise, blur, shadow, etc.

**Figure 1:**
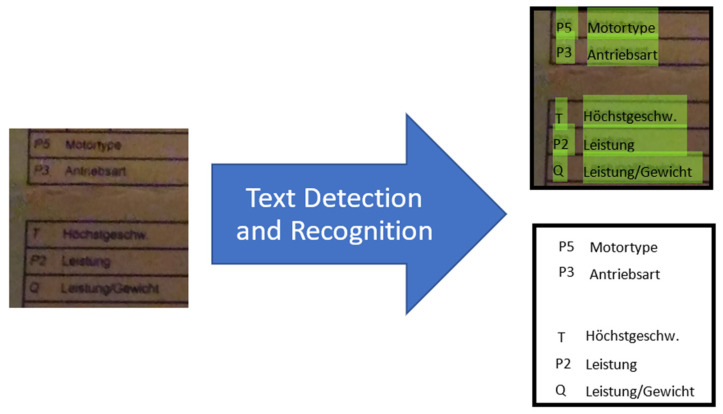
Optical character recognition (OCR).

Therefore, to face those harsh real-world acquisition conditions, one crucially needs a truly robust OCR to recognize the distorted characters and words with an acceptable confidence level for the later meaningful use of those eventually significantly degraded document images. Figure 1 roughly illustrates both the input(s) (i.e., an image or some images) and the respective output(s) of an OCR module. The input document image contains text information. The quality of the input image can be significantly degraded due to harsh environmental conditions during the related acquisition process. The OCR model/system should extract (i.e., detect and classify) text information (i.e., characters and/or words) in its correct position within the input document image in question.

From the image (see Figure 1), two different types of task can be distinguished: rhe first task is that of estimating the “boundary box(es)” that contain text information (one speaks of “boundary box regression”), while the second task is that of recognizing the text within that “boundary box” and mapping it to a corresponding word. The first task is identified as a “regression task”, while the second task is identified as a “classification task”.

**Figure 2:**
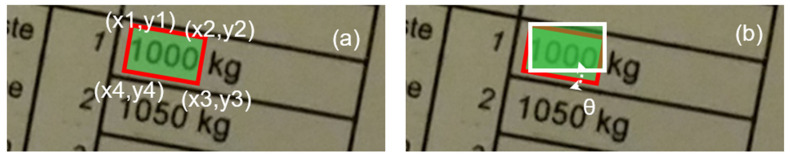
The two different methods to define the text “boundary box”.

Figure 2 illustrates the text detection process (i.e., the two steps) in more detail. First, one detects the related/relevant boundary box. The boundary box, in this case (see Figure 2), due to the current orientation—which is not zero degrees (i.e., not horizontally oriented)—is rotated towards a horizontal orientation, depending on the current/original text position and orientation. Thus, the “boundary box” is defined as a quad polygon (see Figure 2a) or, eventually, a “to-horizontal-rotated” boundary box (see Figure 2b).

Therefore, the text detection loss function based on Figure 2a can be explained through the following formula (see Equation (1) [10]):

(1) $$\min Z=\overset{m}{\underset{j=1}{\sum }}\overset{4}{\underset{i=1}{\sum }}{\left({\bar{x}}_{i,j}-x_{i,j}\right)}^{2}+{\left({\bar{y}}_{i,j}-y_{i,j}\right)}^{2}$$

where Z is the loss function, and the goal is to minimize the value of *Z*. Furthermore, $\bar{x}\;$ and $\bar{y}$ are the expected values. Thus, the vectors x and y are the outputs of the model. The number of points for a box is four. Therefore, *i* varies from 1 to 4. The total number of text boxes within the image is *m*. The total error is calculated by calculating the Euclidean distance between two respective quad polygons using their edge points.

In a second method, we use different information. The text detection loss function based on Figure 2b can be expressed through the following formula (see Equation (2) [10]):

(2) $$\min Z=-\beta \;\log\left(\frac{Area_{intersect}+1.0}{Area_{union}+1.0}\right)+1-Cos\left(\bar{\theta }-\theta \right)$$

where *Z* is the target nonlinear function, and $Area_{intersect}$ and $Area_{union}$ are the intersection and the union, respectively, of the expected text boxes detected within the output of the model. θ is the rotation of the text box as extracted and provided as a further output of the model, while $\bar{\theta }$ is the expected rotation value. The parameter β>1 makes the area precision much more important than the rotation. By selecting large values of β (e.g., 20), this importance of the area precision become even more important compared to the rotation of the boundary boxes.

After the detection of the text bounding box areas, a second/successive module takes care of the recognition of the text contained in those bounding boxes. In this case, the outputs of the model are text elements (i.e., words), which are compared through a loss function with the corresponding expected values. The loss function used for this (text recognition) module/model is a connectionist temporal classification (CTC), which is defined through the formula given in Equation (3) [11]. This loss function is very good when one needs sequences of observations, but the timing and alignment of the observations are not important. For example, in handwritten recognition, the start of a word and where each character occurred can vary, but it should contain the main characters in the right sequence to be accepted as a handwritten word.

(3) $$\min Z=-\log P\left(S|X\right)\;,\;\;P\left(S|X\right)=\underset{c\;\in A\left(s\right)}{\sum }P\left(C|X\right),\;\;P\left(C|X\right)=\overset{T}{\underset{t=1}{\prod }}y\left(c_{t},\;t\right)$$

where *Z* is our target nonlinear function, *S* has the expected sequence values, and *X* is the output sequence of the model. The term P(S|X) is the sum of all possible paths for guessing. For example, if one finds the word “cat” with a sequence length of six (i.e., *T* = 6), the possible sequence values (A(s)) can look like the following examples: “ccaatt”, “c_aatt”, “cca_tt”. The repeated characters are considered as one character, and the symbol “_” is used to escape and go to the next character. The last part of Equation (3), P(C|X), calculates the joint probability of the occurrence of each sequence; for example, “ccaatt”. $y\left(c_{t},\;t\right)$ is the probability of the occurrence of a character in the specified position of *t*—for example, the probability of “a” occurring in the previous example. Meanwhile, T is the maximum length of any given sequence.

The model introduced in this paper uses convolutional neural networks (CNNs). CNNs are a type of deep neural network that mainly use convolution operations, among others, in their many layers. Deep neural networks (DNNs) are essentially multilayer artificial neural networks [12] composed of four main parts or, rather, functional bricks: convolution layers/filters, subsampling layers/filters, activation functions/layers, and “fully connected” neural network layers/blocks. Convolutional neural networks are essentially well-suited for performing a series of complex processing operations, such as estimating inverse filter(s), classification, denoising, contrast enhancement, text recognition, text detection, etc. Previous studies have indeed proven that this type of network can be used for tasks such as denoising [13,14], contrast enhancement [15], deblurring [16,17], text detection [10,18,19,20,21,22], and text recognition [20,23]. Thus, they have enough potential to reliably perform various types of image processing tasks—especially OCR (see Figure 1).

The remaining sections of this paper are structured as follows: Section 2 briefly explains and discusses related works regarding both text detection and text recognition. Our new CNN model is then presented and described in Section 3. In Section 4, our model is comprehensively tested and compared/benchmarked against other models by using the same test dataset for all of them. In Section 5, concluding remarks are formulated, which briefly summarize the quintessence of the results obtained in this research work.

## 2. Related Works

The related works have two different areas, which are considered separately: (a) text detection, and (b) text recognition. Although some models consider these two tasks together, in this paper, our new method addresses them separately.

### 2.1. Text Detection

Text detection, if compared to text recognition, is much harder—especially when trying to find text within natural scene images, as the other objects in the scene normally make the detection harder. Moreover, images that are captured by smartphones have many disturbing artifacts compared to traditional office scanners. Smartphone-captured document images contain blur, focus, shadows, and many other problems that make text detection extremely hard, and often even close to zero.

Overall, one can distinguish three different categories or scenarios for text detection:

(A) Detecting text on scanned images of printed documents that contain no handwriting.
(B) Detecting text on scanned images of printed documents that contain handwriting.
(C) Detecting text on images of natural scenes or on images of printed documents that have been captured by a camera (e.g., smartphone camera).

The text detection model originates from the broader concept of object detection. Object detection by itself is also derived from classification models in general scenes. Two approaches exist for detecting objects in a scene: classical machine learning methods, and deep learning methods [18].

In the first group of methods for text detection, the object (text) is detected through a sliding window, followed by a so-called connected-component-based approach.

In the sliding window, the algorithm is composed of two parts: (a) first, a sliding window through different scales of images, and then (b) a binary classifier that determines whether or not the content of those windows belongs to the text category [19]. The classifier by itself is composed of two parts: The first part extracts features from the image, such as the histogram of oriented gradients (HOG) [20], mean and standard deviation [21], and the edge of the image. The second part is for deciding, based on those features, whether the selected windows belong to the text category.

Three groups of models for detecting text can be distinguished amongst the various deep learning methods. In the first type of model, the text is detected through a so-called boundary box regression analysis. Here, the boundary box of the text is determined as the output of the model. For example, in the “you only look once” (YOLO) method, the boundary boxes are determined using a grid. The algorithm determines the text object within the grid and how the object is shifted on the x- and y-axes with respect to the center of the cell. [22]. In this first type of model, the model is composed of three components: (1) a feature extraction part, which is responsible for extracting the features from the original image, for which pre-trained networks such as ResNet 50, ResNet 101, and VGG 16 with ImageNet can be used [23]; (2) a feature fusion part, where the different extracted features are combined to create combined features; and (3) an output part, where the result of the text detection (i.e., the boundary boxes) is calculated. The nature of the traditional object detection process is different from that of text detection. The main differences are as follows:

(a) Text elements are normally separated, and have no other text elements in their background, but traditional object detection can have multiple objects within one anchor—for example, a person walking in front of a car.
(b) Text can be rotated to the left or to the right, or it can have a curved path. Based on these properties, the text detection models must be extended to support a greater variety of cases (for example, one could take and extend an efficient and accurate existing text detector (e.g., EAST) [10]).

In the second group of methods, the text is instead detected using a so-called semantic segmentation. Here, each pixel is classified into two possible classes: the pixels belonging to the text, and the other remaining pixels. Those pixels that belong to the same text area can be grouped and create a boundary box of text. In this method, instead of regression, classification is used. For creating such a model, different types of semantic classifiers such as UNet can be used [24]. In this latter method, the two different methods of “boundary box regression” and “semantic segmentation” are mixed.

In a third group of methods—the so-called hybrid methods—image segmentation schemes first detect the text areas. After that, the result of the first layer becomes the input of a second model along with the original image. The second layer then uses a single-shot detector (SSTD) [25] or other methods, such as YOLO, to find boundary boxes of text elements within the selected/segmented text areas.

### 2.2. Text Recognition

Text recognition is the process of converting text images into characters or words. Based on the language used, the text recognition is different, and it needs to support all alphabets of that specific language. For example, in the English language, this process needs to recognize 26 lowercase letters, 26 capital letters, 32 ASCII punctuation marks, and the ends of sentences (EOS). Similarly to the first part related to text detection, text recognition is also subject to degradation based on various distorting artifacts, such as brightness, contrast, shadows, focus, blur, poor resolution [26], or environmental conditions such as typefaces/font type [27], text orientation, and text language [28].

Based on these environmental conditions, two main approaches are used to recognize text: (a) traditional/classical machine learning methods [25], and (b) deep-learning-based methods [29]. In the next two subsections, we explore these two approaches further.

#### 2.2.1. Traditional/Classical Machine-Learning-Based Methods for Text Recognition

The traditional/classical machine learning methods can be used to recognize text. This process has three steps: In the first step, the features are extracted using SIFT [30], HOG [20], or other analytical methods. Those features are then processed using traditional classification methods such as SVM [31] and k-nearest neighbors [32]. Finally, the classification output is analyzed based on a visual structure prediction model or a statistical language to remove the misclassified characters [33]. In the traditional methods, one has a bottom-up workflow, whereby the character is first detected and, based on the recognized characters, the words connected/related to the characters can be recognized. For example, one model uses the HOG features and a sliding window technique to extract features; later, the model achieves text recognition by using a pre-trained nearest neighbor or SVM classifier [26].

Amongst the classical methods there also exist some models that can directly recognize the words. For example, one can cite the method by Neumann et Al. [34], who provide a model that has the capability to recognize words using features such as heuristic characteristic detection, aspect ratio, and hole ratio. These features are then used by a classifier such as an SVM. The main limitation of these latter models is their inability to recognize words in degraded/distorted images [35]. Consequently, other approaches have tried to recognized words by using the so-called “template matching” at the word level which, later, is decomposed into the characters. Such an architecture can be found in the works of Almaz’an et al. [35].

#### 2.2.2. Deep-Learning-Based Methods for Text Recognition

Due to the growing power of computers, the possibility of using deep neural networks (DNNs) in the field of character and word recognition is most welcome. The flexibility and power of deep neural networks provide robust models that can accurately recognize text [36].

The first DNN models were created based on feature extraction and then recognizing the characters. After that, those characters, based on non-max suppression information, are merged to form words (see, for example, the work of Wang et al. [37]). In 2013, Bissacco et al. replaced this model with a fully connected network (FCN) and an n-gram technique for character-level recognition [38]. These models were later further developed by involving deep convolutional neural networks using serial softmax classifiers.

In 2016, Jaderberg et al. [39] created a CNN model that could perform word-level recognition. The model was trained and tested on a 90,000-English-word dataset. This model displayed two main problems: (a) it could not recognize words on which it was not trained, and (b) the distortion of characters (due to various distortions, e.g., noise, blur, etc.) could significantly negatively affect the recognition performance.

For solving these problems, several further methods were introduced. The most successful amongst them attempted to consider the relationships between the characters of a word. Therefore, they involved recurrent neural layers (i.e., RNNs) [40] and used the so-called connectionist temporal classification (CTC) loss [41]. These models essentially use a sequence of observations to predict the sequence of labels that include the “blank”. Therefore, in these models, sequence labels have same score even when their alignment and the blanks included are different.

For some time, CTC was used as the main framework for text recognition. However, in 2016, Liu et al. [29] proposed a better model—the so-called spatial attention residue network (STAR-Net) model, which can recognize words even in the presence of spatial irregularity.

In 2014, Bahdanau et al. [42] proposed a method based on attention mechanisms for use in machine translation. Later, this method was combined with CTC to create very good models for recognizing text, such as ASTER [43] and CLOVA [44].

## 3. Our New Method/Model for “Text Detection” and “Text Recognition”

The basic problem formulation is graphically presented in Figure 1, which essentially underscores the core function to be realized by the CNN and/or RNN deep neural models developed in this work. However, to achieve this goal, it has been shown in the relevant literature that a single deep neural network cannot solve the complex problem at hand. Each of the functions “text detection” and “text recognition” is too complex. Thus, we prefer to separate them and solve them in two different models that can then operate in series. The results obtained show that this strategy is a good one.

**Figure 3:**
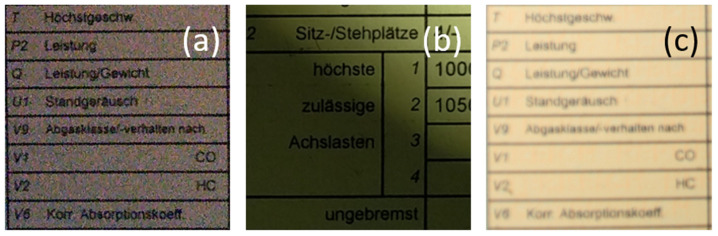
Main distortion problems encountered in document images: (**a**) A document photo, as usually taken from a smartphone with a low amount of light, causing the noise intensity to increase. (**b**) A document image with shadow; some parts are unreadable, whereas some parts are still easily readable with the naked eye; (**c**) A document image with blur; the text in this image is barely recognizable with the naked eye. Source: our own images.

It is important to mention that the document images of interest here are ultimately strongly distorted. Indeed, the primary distorting artifacts that can be found in document images can be categorized into the following three categories (see Figure 3):

- Blur problems, e.g., focus blur, Gaussian blur, or motion blur;
- Noise problems, e.g., salt noise or pepper noise, depending on the image sensor’s sensitivity;
- Contrast problems, e.g., shadows, spotlight, and contrast adjustment.

**Figure 4:**
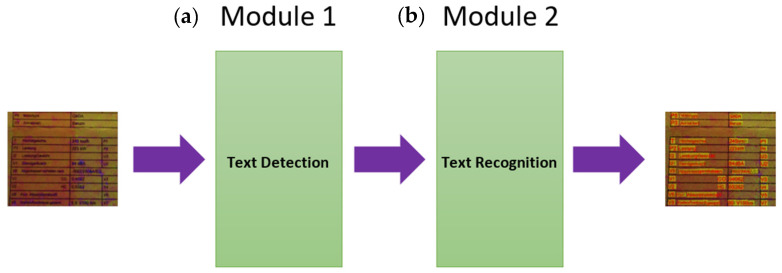
Our new global model, composed of (**a**) text detection (Module 1) and (**b**) text recognition (Module 2).

We developed a model that is robust with respect to the distortions mentioned. Our overall model (see Figure 4) was designed with two modules: (a) a text detection module, and (b) a text recognition module.

The “text detection” module is responsible for detecting the text inside the ”distorted” document image. The result of this model is a set of quad polygons showing the corresponding “text box” boundaries in the input document image. The boundary boxes can be found there in positions (compared to the reference horizontal orientation) that are rotated clockwise (CW) or counterclockwise (CCW), depending on the capturer’s hand position (i.e., how the print document was exposed to the visual sensor). Moreover, the input document image can be distorted by blur (e.g., motion blur and/or focus blur), shadow, contrast issues, noise, etc. The second module in our architecture is responsible for recognizing text within the text boxes identified by the first module. However, the input for this second module must be comprehensively prepared by the first module. Still in Module 1, the boundary boxes that were found by Module 1 are cropped out, and a warping (i.e., geometric rectification) of the cropped image parts (some of this may be in non-rectangular polygon form) into an original rectangular form is carried out. The outputs of this processing are used as inputs of the second module. The second module then determines the contents (text recognition) of the different cropped image parts (i.e., those text boxes that are now rectified into rectangular form). One important remark worth mentioning is that those image parts to be processed by Module 2 are still eventually distorted; Module 1 does not remove the distortions. Indeed, image distortions directly affect the performance of both modules.

### 3.1. Text Detection

Figure 1 shows the general “black box” problem structure of our CNN architecture. As shown above from a comprehensive review of the relevant state of the art, the text detection process is one of the most challenging tasks in computer vision research—especially under harsh distortion conditions. Therefore, it is always necessary to perform various additional preprocessing steps before the CNN model can detect the text bounding boxes with an acceptable final quality.

In the scenarios of relevance for this research, the input images involved in the text detection do not contain just one piece of text, and many of the image samples contain far more. For example, for specific reasons, one can have a document image containing multiple portions of text, some of them even having different sizes and orientations, etc. For such complex document images, one first needs to detect and extract the different text parts (i.e., bounding boxes) contained therein before then individually submitting/inputting them to the next module for text recognition.

Figure 4 shows the detailed architecture of our global model. The input size of the model (see Module 1) is fixed to 512 × 512 with RGB channels. However, maintaining the aspect ratio of any input image is very important. To ensure this, the maximum height or width of the image is resized to fit the 512 pixels while keeping the aspect ratio constant. This resizing process leads to an image containing open areas either on the right-hand side or in the lower region of the input image; these open areas are filled/padded with empty values (i.e., zeros). The output of Module 1 is a list of quad polygons and their respective probabilities of finding optimal bounding boxes. In the next stage, the final list of bounding boxes is structured into a list of image parts containing text, to be further processed for text recognition. Each of them, as identified by Module 1, is cropped/extracted from the original input image and then transformed/resized as an individual input image for the “text detection” module, i.e., Module 2.

**Figure 5:**
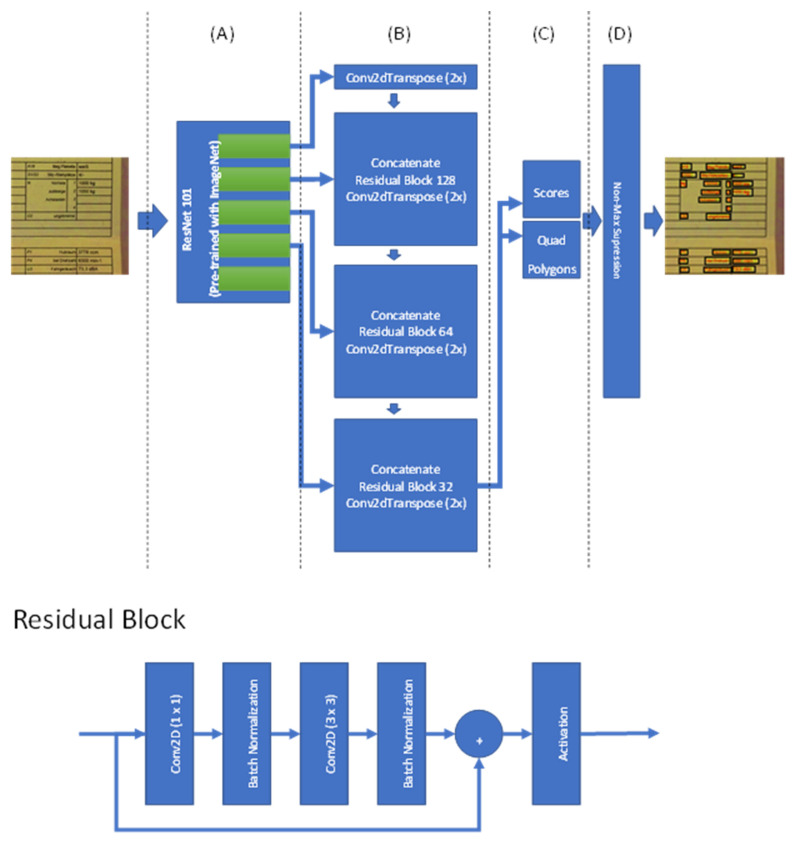
Our detailed architecture for Module 1 indicated in Figure 4. The document detection model contains four parts or sections: (**A**) Feature extraction using pre-trained ResNet 101; (**B**) feature fusion layers using concatenation to merge results and process them using residual blocks; (**C**) output layers, which contain the score values and quad polygons of boundary boxes; and (**D**) a non-max suppression layer.

The text detection model is based on both the so-called “An Efficient and Accurate Scene Text Detector (EAST)” model [10] and the so-called U-Net [45], and it is appropriately customized to be used for our purposes. It contains four main parts: feature extraction, feature fusion layers, output layers and, finally, a non-maximum suppression layer. The feature extraction layers/channels involve a pre-trained ResNet network (ResNet 101). The overall shape of the network is similar to that of U-Net, but the elements of U-Net are created based on ResNet (see Figure 5).

The original EAST model uses a pre-trained PVANet network, but other studies show that it does not provide the required accuracy and precision. Therefore, two main changes were implemented on this model: In the feature extraction part (see Figure 5A), we used ResNet 101. In the feature fusion part of the model (see Figure 5B), we increased the depth of the model without increasing the complexity by introducing residual blocks. The output of the fifth block of ResNet 101 is resized and concatenated with the fourth block and then processed in the residual block. This process is repeated until the output of first block (this output is not used). Finally, the last block of the feature fusion is used to create scores and related quad polygons. The last part of this model architecture is responsible for creating the final quad polygon boundary boxes and creating the final boundary box based on the non-maximum suppression (NMS) algorithm, with a 0.80 overlap threshold.

In Figure 5, the architecture of the residual block is shown below that of the model (see Figure 5). The input of the building blocks is separated into two inputs: the main input, and a shortcut (both have the same value). The main input goes through in a sequence of convolutions with a filter kernel size of 1 × 1, batch normalization, convolution with a filter kernel size of 3 × 3, and further batch normalization. Finally, the output of the main branch is added to the shortcut value and, after passing through the activation function, creates the final output of our residual block. The number of filters is a parameter that is defined through each block of the model fusion segment (see Figure 5B).

Here, convolutional transpose layers are used to increase the size of the images. This is done by choosing a kernel size of 4 and a stride of 2. All convolutional layers used in Figure 5 have ReLU activation functions. ReLU activation functions [46] are known to show an outstanding convergence rate compared to other activation functions.

Our designed model (see Figure 5) was trained using augmented document image samples, which were artificially modified, i.e., rotated, scaled, and cropped. The sample data used were derived from the following datasets: International Conference on Document Analysis and Recognition (ICDAR) 2013, ICDAR 2015, and our own created dataset.

**Figure 6:**
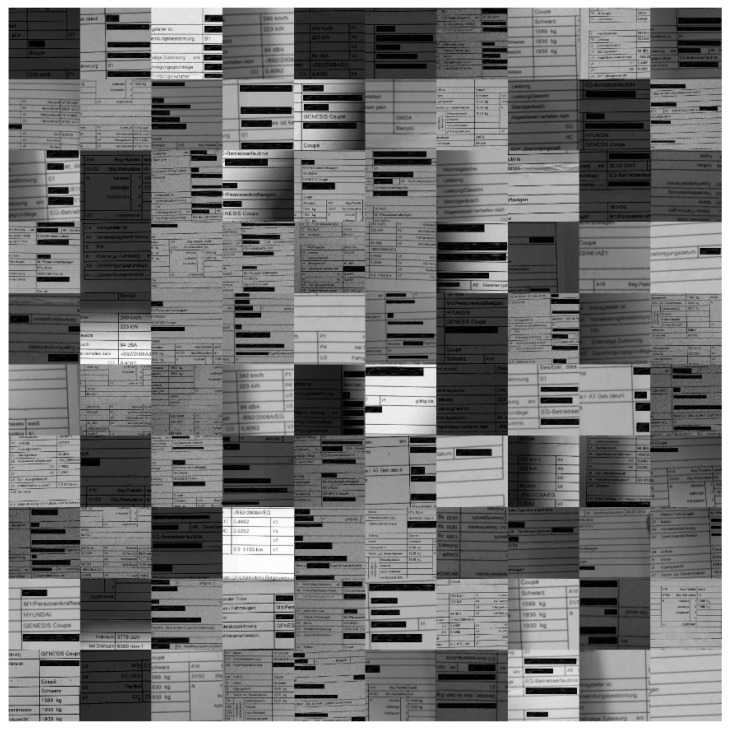
These are 100 **“very bad quality”** representative sample data (an extract from a much bigger dataset). Note: since the images contain some personal data, those parts are covered by black rectangles for privacy reasons.

**Figure 7:**
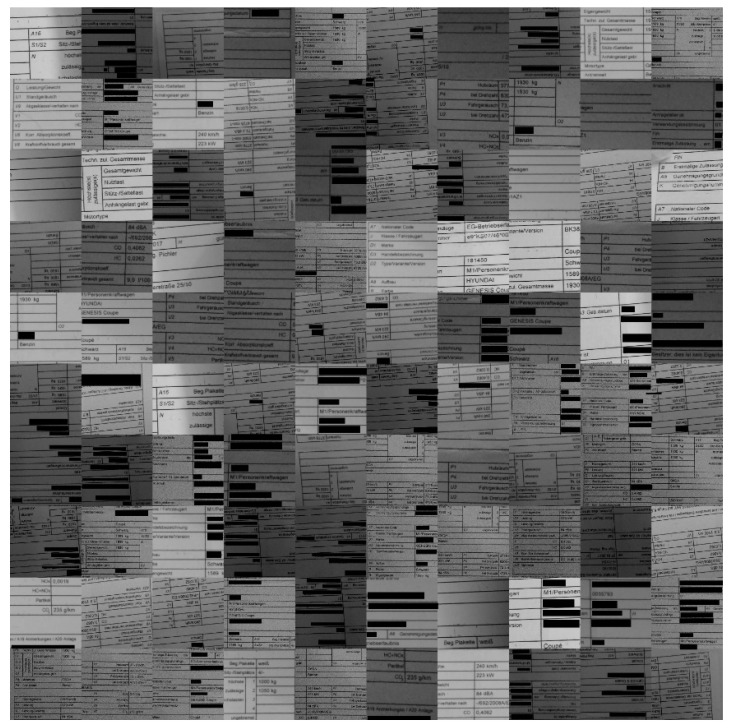
These are 100 **“bad quality” sample** data extracted from our own dataset (an extract from a much bigger dataset). Note: since the images contain some personal data, those parts are covered by black rectangles for privacy reasons.

**Figure 8:**
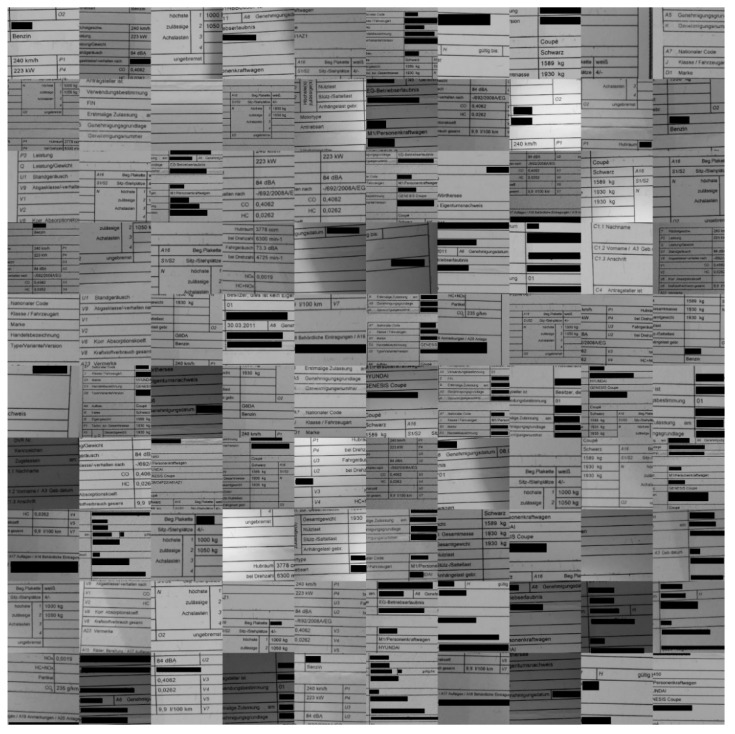
These are 100 **“middle quality” sample** data extracted from our own dataset (an extract from a much bigger dataset). Note: since the images contain some personal data, those parts are covered by black rectangles for privacy reasons.

**Figure 9:**
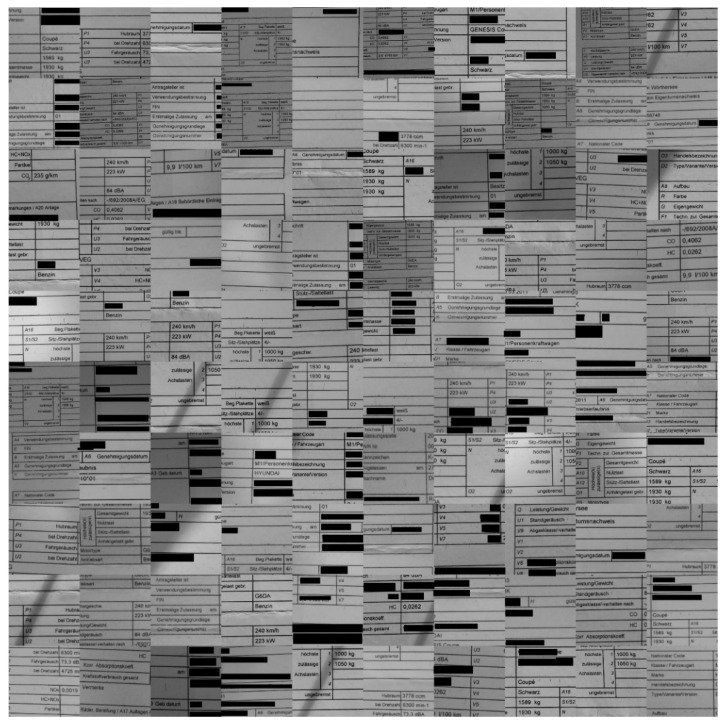
These are 100 **“good quality”** sample data extracted from our own dataset (an extract from a much bigger dataset). Note: since the images contain some personal data, those parts are covered by black rectangles for privacy reasons.

**Figure 10:**
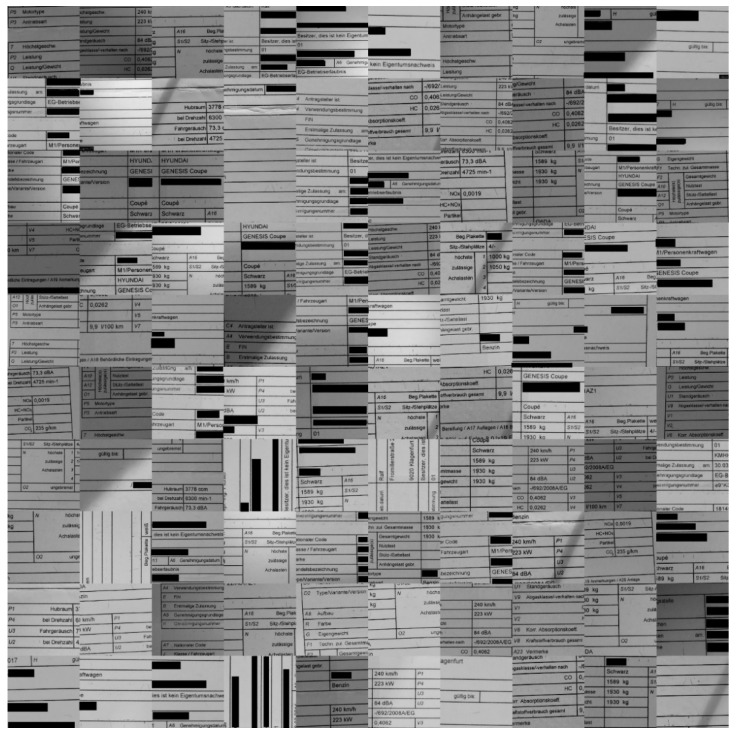
These are 100 **“very good quality**” sample data extracted from our own dataset (an extract from a much bigger dataset). Note: since the images contain some personal data, those parts are covered by black rectangles for privacy reasons.

Our own created dataset presented in this section consists of document images obtained by our team under harsh acquisition conditions (i.e., using cameras) (see the illustrations presented in Figure 6, Figure 7, Figure 8, Figure 9 and Figure 10). The main reason for the harsh conditions was to provide strongly distorted images (i.e., contaminated by a mixture of distortions) in order to better stress-test the robustness of the models involved in the comprehensive benchmarking (i.e., our new model and a selection of the best models from the relevant literature).

The sample images of the benchmarking dataset collected under realistic harsh conditions were sorted based on the text recognition performance of Tesseract (open-source OCR software) on them. Five different categories were defined: (1) very good images on which the OCR can detect the words with an accuracy in the range of 90–100%(see samples in Figure 10); (2) good images on which the OCR can detect the words with an accuracy in the range of 80–90% (see samples in Figure 9); (3) medium images on which the OCR can detect the words with an accuracy in the range of 70–80% (see samples in Figure 8); (4) bad images on which the OCR can detect the words with accuracy in the range of 60–70% (see samples in Figure 7); (5) and very bad images on which the OCR can detect the word with accuracy lower than 60% (see samples in Figure 6).

As they were the most strongly distorted, categories 4 and 5 (see Figure 6 and Figure 7, respectively) were those involved in the hard benchmarking processes for both text detection and text recognition.

As the numbers of sample data for training and testing were relatively small, augmentation techniques were used to significantly increase the number of samples during the training process. The following augmentation techniques were performed: randomly scaling, rotating, and cropping the source images.

The evaluation indices used for the “text detection” endeavor as usual, were precision (P) and recall (R), which are defined as follows:

(4) $$P=\frac{TP}{TP+FP}\;\;\;,\;R=\frac{TP}{TP+FN}\;\;\;\;$$

where TP is the correct detection (i.e., true positives), FP is the wrong detection (i.e., false positives), and FN is the missing detections (i.e., false negatives).

### 3.2. Text Recognition

**Figure 11:**
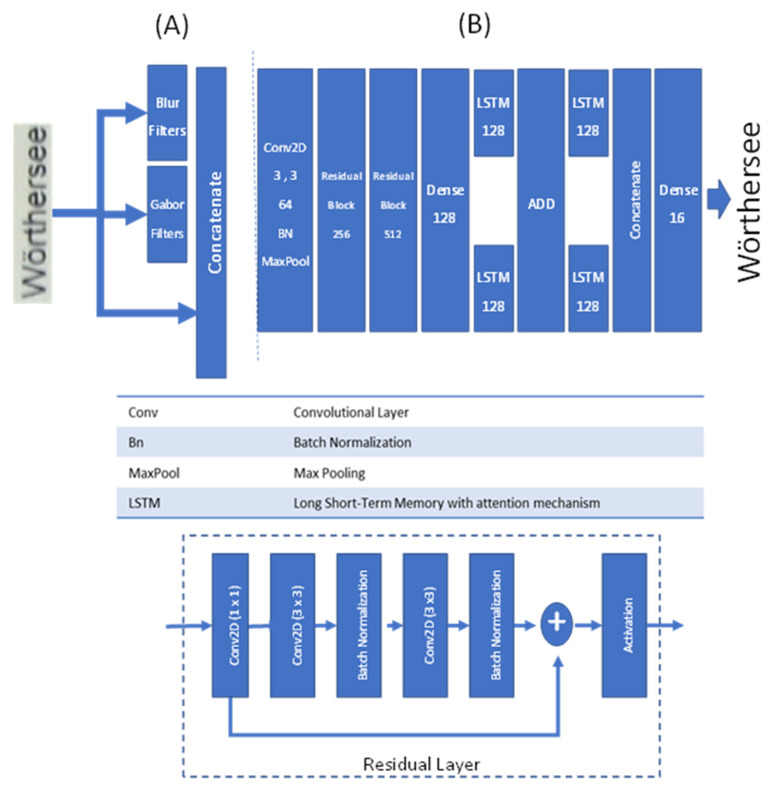
Our new text recognition architecture for Module 2 (of the architecture shown in Figure 4): This module starts with (**A**) preprocessing layers, and continues with (**B**) feature extraction of the text image. In the middle, the model uses residual layers and LSTM with an attention mechanism to perform feature fusion. Finally, the model uses those features to find/determine a word.

The second module of our global architecture shown in Figure 4 is described in Figure 11. This module is responsible for text recognition. The main challenge faced by this module is related to the large number of possible words to be recognized, which are not always the same, and depending on language, the maximum length of words and the number of characters used in that word can vary significantly. Then, the extracted image portions containing text from the first module are scaled to the height of 32 pixels. The width can be extended up to 32 × 16 pixels. The maximum word length of 16 characters covers almost 95% of German-language words. This enables the model to be suitable for most German-language text recognition, as most of the text in various documents has a smaller or equal size. The input image is provided via three (color) channels. The output of this module is a sequence of German alphabet characters, digits, and punctuation.

The text recognition model contains three separate parts: The first part comprises the preprocessing layers. The feature extraction preprocessing layers/channels contain different well-known filters, such as blur and Gabor filters (see Figure 11). Compared to the original convolutional recurrent network (CRNN) [41] model, the following changes are introduced: (1) preprocessing layers, (2) introducing residual layers, and (3) replacing LSTM layers with LSTM with attention mechanism. In the first part of model (See Figure 11A) the preprocessing layers and the “Gabor filter” are added. These new preprocessing filters help support the model in focusing on aspects of the input image that are critical and relevant for the classification task. The second part of model (See Figure 11B) uses a residual block of ResNet-like architecture as a set of feature extraction layers. The main reason for choosing this architecture (for the second part) is to provide a deeper network with increasing convergence speed of convergence [47]. Indeed, this enables the network to detect character blocks very easily. The third part of the model contains a feature fusion part. In this part of the model, the extracted information is fused together to provide more sophisticated features. In the last part, we have our output layers, which provide our desired outputs as the final output of the model. Again, to improve the quality of text detection, the LSTM layer is replaced by LSTM with attention mechanism [48]. This proves (as confirmed by our final results) to be very useful and efficient for better text recognition.

## 4. Models’ Training and Comprehensive Testing (for Both “Text Detection” and “Text Recognition”), Results Obtained, and Discussion of the Results Obtained

The model training strategy is the most challenging part. Indeed, it is necessary to address and include different types of input image distortions (e.g., blur, noise, and contrast issues) in the training datasets. Both quality and quantity of the training samples can help to adjust/tune the weights in the models. Each submodule converges to the best parameter settings corresponding to each submodule’s respective defined task. The training datasets comprise six datasets: three of them are used for “text detection” (see the first module of Figure 4), and the other three are used for “text recognition” (see the second module in Figure 4).

The training of Module 1 for “text detection” is structured as follows:

(a) ICDAR 2013 dataset: This dataset contains 229 training images and 233 testing images with word-level annotation.
(b) ICDAR 2015 dataset: This dataset contains 229 training images and 233 testing images with word-level annotation.
(c) Our own dataset: The samples in this dataset were gathered by our team. It has 456 images, 270 of which are used for training. These images are unique, as they capture and present different kinds of real-world distortions that can take place in document images taken using smartphone cameras. Figure 6, Figure 7, Figure 8, Figure 9 and Figure 10 show illustrative parts of our own dataset.

The first module, Module 1, was trained as follows: The ICDAR 2013 dataset was used as the first training dataset. After finishing the training with the first dataset, the trained module was trained again using ICDAR 2015. Finally, it was trained with our own collected/created dataset. In this way, the training knowledge was well transferred, resulting in a robust model.

**Figure 12:**
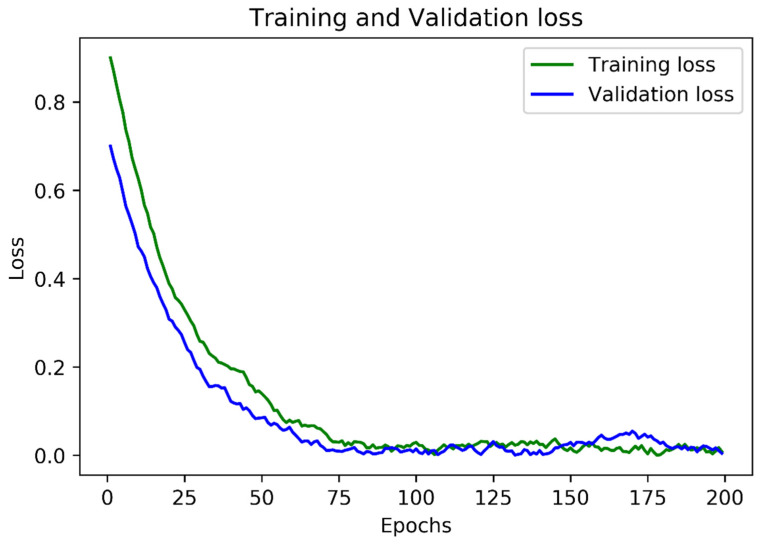
The evolution of both training and validation performance over the epochs; here, we show the first 200 epochs.

In the first module, the ADAM [49] optimizer was used to train the model end-to-end. For faster learning, we sampled 512 × 512 crops from images to form batches of 128 images. The learning rate of ADAM started at 1 × 10^−3^, decreased at a decay rate of 0.94 after 100 epochs to improve the loss value, and finally stopped at 1 × 10^−5^. The network was trained until it reached 800 epochs. Figure 12 shows the training of the first module using the loss function explained in Equation (2) while using the ICDAR datasets for training and validation.

**Figure 13:**
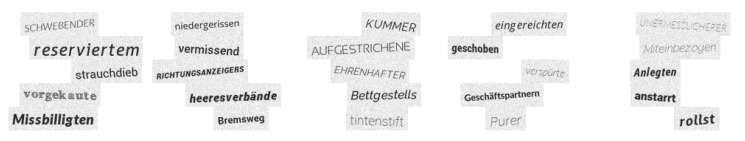
Sample German words generated by our Python module for training the text recognition for German words.

For the second module, there are also three datasets, as follows:

(a) MJSynth dataset: This dataset contains 9 million images covering 90,000 English words [50].
(b) Generated German dataset: This dataset contains 20 million images covering 165,000 German words. The images were created using an author’s written module in Python and the dictionary used is taken from aspell.net, which contains many open-source dictionaries for spell checking. The generated data are synthetic, based on words from the dictionary and generated by our Python module (see Figure 13).
(c) Our own dataset: The samples of this dataset were gathered by our team. It has 4560 word images. These images are unlike those of the other datasets, as they contain real-world distorted images, some of them strongly distorted; these are not synthetic data. Figure 6, Figure 7, Figure 8, Figure 9 and Figure 10 show illustrative parts of our own dataset.

The second module, like the first module, was trained as follows. To start, the MJSynth dataset was used as the first training dataset. After finishing the training with the first dataset, the trained module was trained again using the “Generated German dataset”. Finally, it was trained with our own dataset. In this way, the training knowledge was well transferred to generate a very robust model.

**Figure 14:**
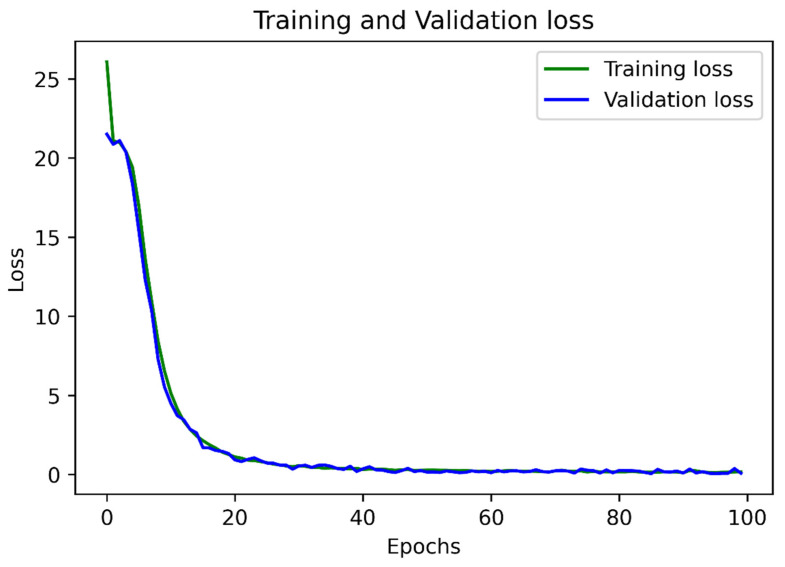
The evolution of both training and validation losses over the first 100 epochs.

In the second module, the ADAM [49] optimizer was used to train the model end-to-end. For faster learning, a batch size of 512 images was selected. The learning rate of ADAM started at 1 × 10^−2^, decreased to one-tenth after three attempts failed to improve the loss value, and finally stopped at 1 × 10^−5^. The network was trained until its performance stopped improving after 10 attempts. Figure 14 shows the training of the second module using the loss function explained in Equation (3) while using the MJSynth dataset for training and validation.

The computing system used for implementing our models was a PC with the following settings: Windows 10 Pro with the latest patches, Intel Core i7 9700K CPU, double Nvidia GeForce GTX 1080 TI with 8 GB RAM GPU, and 64 GB RAM. The training of the first module took approximately 23 h with all datasets, and the training of the second module took about 48 h using all datasets.

### 4.1. Performance Results of Module 1 for Text Detection

In this subsection, we compare the performance of our Module 1 with a selection of well-known text detection methods, as presented in several recent papers from the relevant literature. Some of these methods from the literature are analytical, while some of them use CNNs.

**Figure 15:**
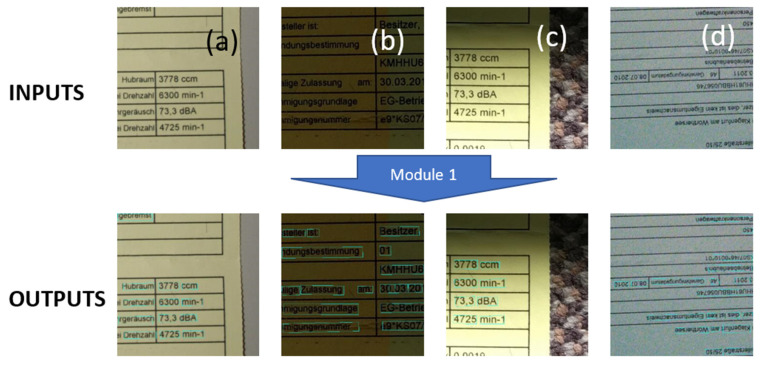
Samples of detected text bounding boxes obtained using our Module 1 as shown in Figure 5. The detected text boxes are marked with colored rectangles: (**a**) The text detection under normal conditions, i.e., very small or almost no distortion. (**b**) The text detection with contrast problems. (**c**) The text detection with shadow problems. (**d**) The text detection with noise and rotation problems.

In Figure 15, we present examples of text detection test results by our Module 1. As we can see in these sample images, good text detection can be achieved even for very badly distorted input document images contaminated by issues such as noise, contrast, shadows, and blur (e.g., motion blur or focus blur).

The evaluation indices used for the “text recognition” endeavor were the word- and/or character-recognition-related precision (P), and the word- and/or character-recognition-related recall (R), respectively, which are both defined in Equation (4).

In Figure 15a, the reference input is a “good”-quality document image, and the text is clearly detected. Here, there are ideal conditions, where the image has good light, no blur, and no shadow problems. The other images (i.e., (b–d)) were degraded under real-world conditions; this illustrates very clearly that our model can detect the text even under hard conditions.

In the next step, the model was compared to the state-of-the-art models of text detection (see Table 1).

The results presented in Table 1 clearly show that most “text detection” models produce a very high number of errors and, therefore, cannot be used for reliable text detection for subsequent reading (i.e., text recognition) by Module 2. Although still weaker compared to our Module 1, the only previous method with an acceptable performance is the one using CLRS [55]. Among all of the results, our model (Module 1) shows the best performance, with 95.4% precision and 96.8% recall. The test dataset was our dataset with different qualities.

Table 2 shows how the different levels of distortion, as expressed by the five document-image quality levels, impact the performance of our novel “text detection” model with respect to the precision and recall metrics. It is clear (see Table 2) that the decrease in document-image quality results in a reduction in the OCR performance.

### 4.2. Performance Results of Module 2 for Text Recognition

In this subsection, we provide and briefly discuss a set of illustrative performance results of Module 2.

Regarding the evaluation metrics, two metrics that are generally used for assessing text recognition performance are considered: the character-recognition accuracy (CRA), and the word-recognition accuracy (WRA). The CRA is the percentage of the total number of characters that are recognized correctly, and the WRA is the percentage of the total number of words that are recognized correctly. The related studies show that the WRA metric is generally used to compare the text-recognition performance of various schemes [29,43].

**Figure 16:**
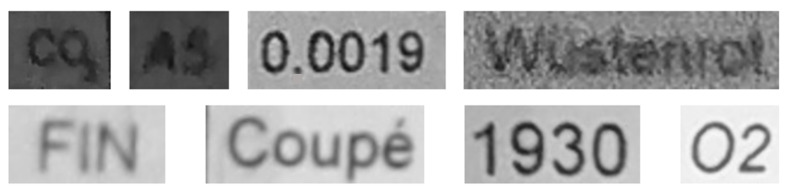
Samples of text recognition inputs obtained by using our Module 1, as shown in Figure 5. The detected text images were cropped from the input image and then used as inputs of the second module to recognize the text information contained therein.

Figure 16 shows some selected inputs of Module 2. As we explained previously, the second module performs text recognition under hard conditions. Indeed, Figure 16 clearly shows some hard recognition cases for which the text is easily recognized using our model.

**Figure 17:**
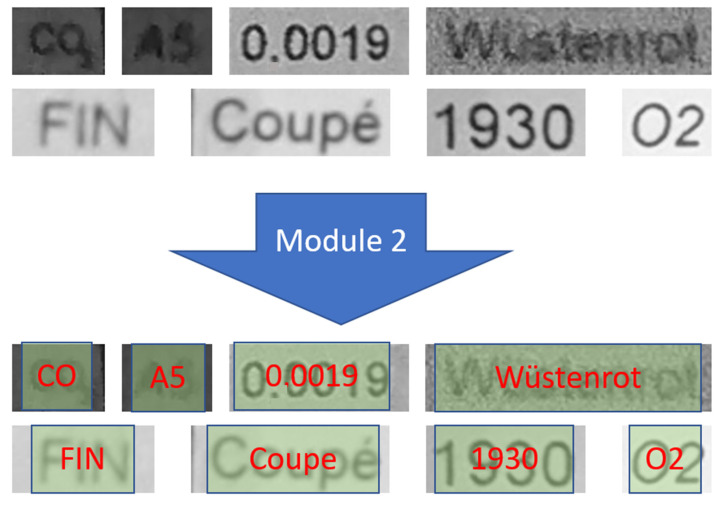
Sample of text recognition using Tesseract (open-source OCR system) and our novel OCR model. As we can see, most of the text samples are recognized in these images, but Tesseract cannot read them.

In Figure 17, some examples of different input document images for Module 2 are shown. All of the text contained in those badly distorted images was easily recognized using Module 2, as can be seen in Figure 17. A comprehensive performance comparison with most relevant related works from the recent literature is provided in Table 2. All of those different models were tested on our reference datasets (represented by Figure 6 and Figure 10 as illustrative samples).

The results presented in Table 3 clearly show that most of the competing “text recognition” models produce a very high number of errors and, therefore, cannot be reliably used for robust text recognition as an alternative to our Module 2. Our Module 2 is clearly superior and much more robust compared to all other models involved in the benchmark, as underscored by Table 2. Although still significantly weaker compared to our Module 2, the only previous methods/models with a relatively acceptable performance were the ones using ASTER [43] and CLOVA [44]. However, our new model remains significantly superior to all of them.

Table 4 shows the effects of different document-image quality levels on the precision and recall metrics with respect to the “text recognition” performance. It is clear that by decreasing the quality of the document images, the text recognition also becomes lower (i.e., it is reduced).

## 5. Conclusions

In this study, we developed a new deep learning architecture model to reliably detect and recognize text even in strongly distorted (e.g., by blur, noise, shadows, contrast issues, etc.) document images using different European language dictionaries. Our new model is a very robust OCR system model.

This demonstrated robustness was achieved by combining two different modules in sequence. Each module was trained by well-prepared datasets that were tuned and specialized for their specific tasks. This task separation (text detection on the one hand, and text recognition on the other) significantly contributes to the outstanding performance achieved by our global model.

The first module (i.e., Module 1) of our global model outperforms the best competing models from related works with respect to text detection, by at least 13% (see Table 1). Meanwhile, the second module (i.e., Module 2) outperforms the best competing models with respect to text recognition by at least 7.5% (see Table 3).

In conclusion, our developed global model significantly outperforms all other schemes, as illustrated by two comprehensive extensive benchmarks. Thus, its clear superiority is sufficiently underscored.

## Figures and Tables

**Table 1:** The test results of a comprehensive benchmarking of our model (for text detection) against selected state-of-the-art models.

Method	Recall	Precision	FPS (Frames per Second)
SegLink [51]	76.8	73.1	-
EAST [10]	70.8	79.2	13.2
PixelLink [52]	81.7	80.7	7.3
TextSnake [53]	85.3	81.5	1.1
SegLink++ [54]	80.4	82.5	7.1
CLRS [55]	70.7	90.1	1.1
TextField [56]	83.9	83.1	1.8
PSENet [57]	84.5	85.9	1.6
DB-ResNet-50 [58]	87.3	81.7	26
Our model (First Module)	**96.8**	**95.4**	**4**

**Table 2:** The test results of a comprehensive benchmarking of our model (for text detection) with different document-image quality levels of our test dataset.

Image Quality vs. Precision and Recall	Very Good	Good	Middle	Bad	Very Bad
Precision	100%	100%	98.9%	98.3%	90.1%
Recall	100%	100%	99.1%	98.3%	89.8%

**Table 3:** These results compare the performance of our model (for text recognition) with that of relevant selected state-of-the-art models under the same conditions.

Method	WRA	CRA
CRNN [41]	85.2	73.1
RARE [59]	84.81	79.2
ROSETTA [40]	86.1	80.7
STAR-Net [58]	86.6	81.5
CLOVA [44]	88.2	82.5
ASTER [43]	86.9	90.1
Our model	**98.21**	**97.51**

**Table 4:** The test results of a comprehensive benchmarking of our model (for text recognition) with different document-image quality levels of our test dataset.

Image Quality vs. WRA and WCA Performance	Very Good	Good	Middle	Bad	Very Bad
WRA	98.32	98.29	91.81	92.43	81.53
WCA	99.51	98.69	95.06	92.13	84.64

## Data Availability

Not applicable.
